# Urine cultures in a long-term care facility (LTCF): time for improvement

**DOI:** 10.1186/s12877-018-0909-x

**Published:** 2018-09-20

**Authors:** J. Haaijman, E. E. Stobberingh, L. W. van Buul, C. M. P. M. Hertogh, H. Horninge

**Affiliations:** 1 River Region Elderly Care Centers (SZR), Burgemeester Meslaan 49, 4003CA Tiel, The Netherlands; 2 Faculty of Health, Medicine and Life sciences, Department of Medical Microbiology, Maastricht University Medical Center (MUMC), School of Public Health and Primary Care (CAPHRI), Maastricht, The Netherlands; 3 Amsterdam Public Health Research institute and Department of General Practice & Old Age Medicine, VU University Medical Center, 1081 BT Amsterdam, The Netherlands

**Keywords:** Urine cultures, Long-term care facilities, Urinary tract infection, Antibiotics

## Abstract

**Background:**

Urinary tract infections (UTIs) are the most prevalent infections in long-term care facilities (LTCFs). Numerous studies have described the problem of inadequate UTI diagnosis and treatment. We assessed the role of urine cultures in the diagnosis and treatment of UTIs in a LTCF.

**Methods:**

In a 370-bed non-academic LTCF a retrospective assessment of antibiotic (AB) prescriptions for UTIs and urine cultures was performed from July 2014 to January 2016. The reasons why physicians, including 11 nursing home physicians and 2 junior doctors, ordered urine cultures were recorded using questionnaires.

**Results:**

During the study period, 378 residents were prescribed 1672 AB courses; 803 were for UTIs. One hundred and fifty-five urine cultures were obtained from 135 residents; 66 of these cultures were performed on the same day as ABs were prescribed (8% of all prescriptions for UTI), while 89 were not. There was a discrepancy between the actions that seemed logical based on the culture results and the actions that were actually taken in 75% of the cases. In these cases, initial AB treatment was not adjusted when the isolated microorganism was resistant to the AB prescribed, the urine culture was positive and no ABs had previously been administered, or ABs were prescribed and no microorganism was isolated.

The most frequent reason for ordering a urine culture was to confirm the diagnosis of a UTI.

**Conclusion:**

In the majority of patients, AB therapy was not adjusted when the urine culture results suggested it may be appropriate. The physicians were erroneously convinced that UTIs could be diagnosed by a positive urine culture.

**Electronic supplementary material:**

The online version of this article (10.1186/s12877-018-0909-x) contains supplementary material, which is available to authorized users.

## Background

Antimicrobial resistance (AMR) is an increasing threat to the effective treatment of patients with bacterial infections worldwide [1]. The amount of antibiotics (ABs) that are prescribed and the degree of AMR that develops are directly related [2]. In long-term care facilities (LCTFs), the number of AB prescriptions ranges from 50 to 200 annually per 100 residents [3–5]. Many studies have described the incorrect usage of ABs in LTCFs, which is partly due to incorrect diagnosis and partly due to incorrect AB choices [3–9]. Most ABs used in LTCFs are prescribed for the treatment of urinary tract infections (UTIs) [4, 5].

Diagnosing UTIs in frail elderly individuals is complicated; a positive urine culture is frequently considered the gold standard [10], but a positive urine culture implies the presence of bacteriuria only and not a symptomatic UTI [10]. Asymptomatic bacteriuria (ASB) frequently occurs in LTCF residents. The prevalence of ASB ranges from 25 to 50% in women and from 15 to 40% in men [11–14]. ASB can co-exist with the presence of signs and symptoms that are often unrelated to UTIs [10, 14–16].

The decision regarding whether to diagnose and treat these signs and symptoms as a UTI is often based on the observations of the nurse who considers that the patient is “not being herself today “and asks the doctor to come and see the patient. The physician makes the decision to prescribe AB therapy based on the patient’s symptoms, additional laboratory results if they are considered relevant, and his clinical experience. Whether the results of urine cultures contribute to the decision process remains unknown.

In the current study we aimed to answer the following questions for our LTCF:

1. How often are urine cultures performed on the same day just prior to administration of an AB for a UTI?
2. What is the effect of urine culture results on treatment choices for UTIs?
3. For what reasons do physicians order urine cultures?

## Methods

To answer the first two questions, we retrospectively analysed the empiric AB prescriptions for UTIs, the results of urine cultures ordered and the actions taken after the urine culture results returned according to the corresponding patient records (the notes of physicians and nurses). We assessed whether the urine culture results met the criteria for reasons for action. We defined the criteria for reasons for action based on urine culture results as follows: i) the isolated uropathogen was resistant to the AB prescribed; ii) no AB therapy was prescribed, and the result of the urine culture was positive; iii) AB therapy was prescribed, and the result of the urine culture was negative.

To answer the third research question, we sent out questionnaires asking the physicians why they had ordered urine cultures.

### Setting

The study was performed in a 370-bed LTCF in the Netherlands in a non-academic setting, with 45 beds for short-stay geriatric rehabilitation, 150 for somatic residents and 175 for psychogeriatric residents.

All physicians providing medical care in this LTCF participated in the study, including 11 nursing home physicians and 2 junior doctors. The mean age of the physicians was 47 years (age range: 26–62 years). The physicians obtained their medical and vocational training at 5 different universities, and the mean number of years of experience in a LTCF setting was 15 years (range: 0 to 34 years).

### Retrospective analysis of urine cultures and AB therapy

First, a list of all ABs with an Anatomical Therapeutic Chemical (ATC) Classification System code J01 prescribed from 1 July 2014 to 1 January 2016 was retrieved from the electronic prescribing system (EPS). The ATC J01 is the code for systemic antibacterial drugs.

Next, the indications for AB prescriptions on the list were retrieved from the corresponding electronic patient records (information from the notes of the physicians and nurses was used; there was no systematic coding of diseases or reasons for doctor encounters, such as the International Classification of Primary Care (ICPC) or International Statistical Classification of Diseases version 10 (ICD-10), available in the electronic patient records). Only those ABs prescribed for (putative) UTIs were selected and grouped by patient to identify recurrent prescriptions during the study period. Recurrent UTIs were defined as the occurrence of more than three UTI episodes in the previous year. ABs prescribed for long-term prophylaxis were excluded.

The urine samples for culture were brought to the nearby hospital and were then transported once daily to a medical microbiological laboratory a distance of 35 km away.

Microbiological analysis of the urine cultures was performed according to standard methods [17–19]. A urine culture was considered positive in cases with >10e4 colony-forming units (cfu) of gram-negative uropathogens/ml. We focused only on gram-negative species. The isolation of more than two different microorganisms was considered contamination. AB susceptibility testing was performed according to the Eucast guidelines [17–19].

The results of the cultures were provided to doctors in three different ways: they were added to the electronic patient records and a printed version was delivered to both the physician’s mailbox and the ward’s mailbox.

By comparing the results of the urine cultures with the AB prescribed (on the same day as the urine sample was obtained and within a week after the culture results were reported), we could answer the second question regarding the effect of the urine culture results on the treatment choices for UTIs. For that purpose, the records of all residents with urine culture results were reviewed to assess the actions taken, i.e., the initiation or discontinuation of or change in AB treatment and the reasons why.

The turnaround time, i.e., the time between obtaining the urine samples and receiving the results, was recorded.

All data were analysed descriptively.

### Assessment of the reasons why urine cultures were ordered

To answer the third question, we used a questionnaire that was completed regarding the indications and reasons for which physicians had ordered urine cultures between August 1, 2015 and April 1, 2016 (Fig. 2). More than one reason was possible, and an option was included for reasons not listed in the questionnaire. The Questionnaire is available as Additional file 1.

Two nurses sent questionnaires to the physicians when urine cultures were ordered and sent a reminder within one week when a questionnaire was not returned.

The data were analysed descriptively.

## Results

### Retrospective analysis of urine cultures and AB therapy

During the study period between July 2014 to 1 January 2016, the total number of AB prescriptions with the ATC code J01 was 1672; of these, 869 were excluded because they were prescribed for indications other than UTIs, resulting in 803 AB courses prescribed for the treatment of (putative) UTIs in 378 patients. Of these patients, 299 were females (79%), and 79 were males (21%). There were 144 psychogeriatric patients (38%), 159 somatic patients (42%) and 75 patients admitted for a short stay to a geriatric rehabilitation ward (20%). Thus, there were 197.741 resident-days and 4.1 AB prescriptions for UTIs per 1000 resident-days. Of the 378 patients, 191 (50.5%) received one prescription, and 187 (49.5%) received more than one (with a maximum of ten).

**Fig. 1:**
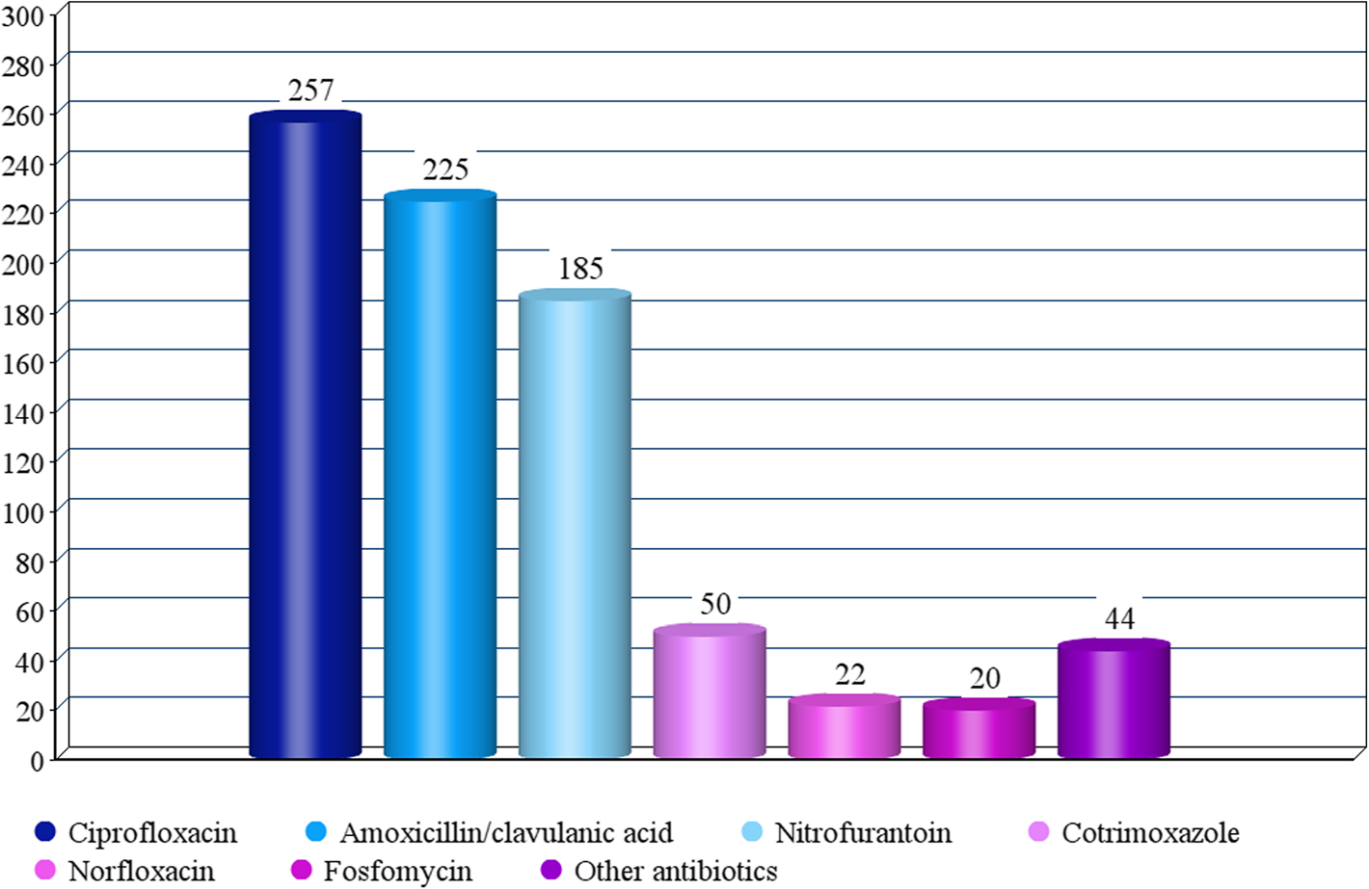
Eight hundred three antibiotic prescriptions for UTI

Figure 1 depicts the 803 AB prescriptions. There were 257 (32%) prescriptions of amoxicillin/clavulanic acid, 225 (28%) of ciprofloxacin, 185 (23%) of nitrofurantoin, 50 (6%) of cotrimoxazole, and 22 (3%) of norfloxacin.

A total of 159 urine cultures were ordered for 135 residents. More than 1 culture was performed for 22 residents (2 cultures in most residents, and 3 cultures in 2 residents). The culture samples were obtained on the same day as empiric ABs were prescribed (and before the AB was given to the patient) in 66 of 803 prescriptions (8%).

In ninety-three cases, a urine culture was performed, and no antibiotics were prescribed before the results of the culture were obtained. Thirty-six (39%) of these 93 cultures were ordered because of recurrent UTIs.

The turnaround time for the culture results ranged from three days for cultures with negative results or contaminated cultures to seven to twelve days for cultures with positive results**.**

**Fig. 2:**
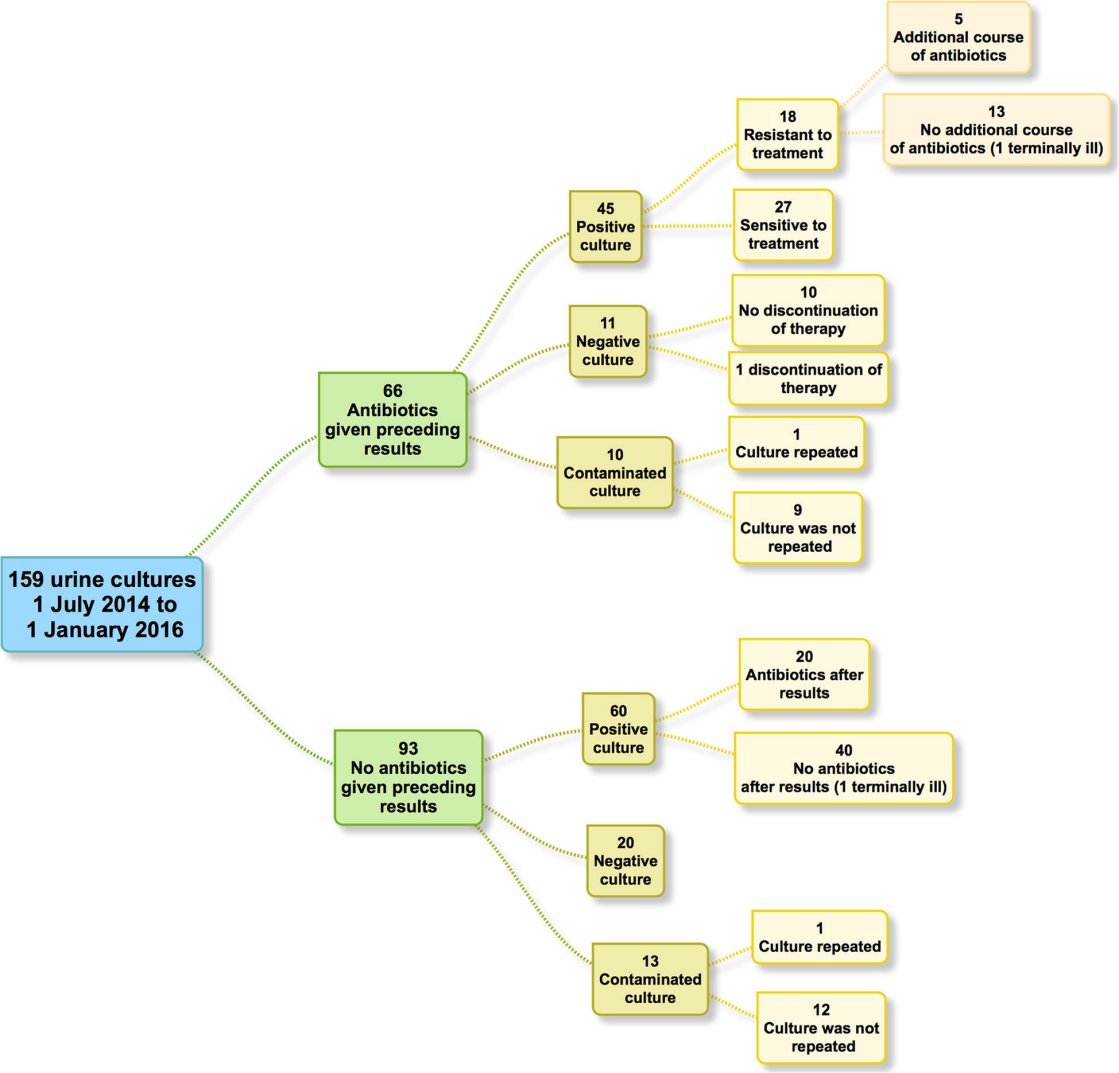
One hundred fifty-nine urine cultures (1 July 2014 to 1 January 2016)

Figure 2 shows the results of all the urine cultures and the actions taken by the physicians. Of the 159 cases in which a urine culture was performed, empiric AB treatment was initiated on the same day that the urine sample was collected in 66 cases (42%). Of the 66 cases in which the resident received ABs prior to the culture results being reported, 45 cultures were positive (68%). Of these cases, 18 (40%) had a microorganism isolated on urine culture that was resistant to the prescribed AB. In five of these 18 patients (28%), the AB therapy was adjusted, and in 13 (72%), not adjustments were made.

Of the 66 cases in which empirical treatment was started before the culture results, 11 (17%) had negative urine cultures; in one case, the treatment was discontinued. Of the 10 patients in this group who had contaminated cultures, a culture was repeated in one patient.

Of the 93 (58% of 159 residents) cases in which no AB therapy was initially administered, 60 urine cultures (65%) were positive. AB therapy was prescribed in 20 (33%) of these 60 patients, and 40 (67%) received no treatment. In this group of 93 cultures, 13 were contaminated, and one was repeated.

In 112 (70%) cases, the culture results suggested reasons for action, such as the initiation or discontinuation of or change in AB therapy or repeated culture because of contamination. In 84 (75%) of these 112 cases, no action was taken. In 28 cases (25%), actions were taken according to the culture results.

For two of the residents, no action was taken based on the culture results. In one resident, no further AB treatment was prescribed as part of advanced care planning at the end of life. The other resident was terminally ill when the results were received: therefore, no action was taken. In all other residents, neither the culture results nor the reasons for not taking action were mentioned in the records.

Positive culture results are depicted in Table 1. *E. coli* and Proteus mirabilis were the most frequently isolated microorganisms, and they were cultured in 38% and 30% of samples, respectively. *E. coli* showed the highest resistance to amoxicillin (70%) and the lowest resistance to nitrofurantoin (6%). Proteus showed the highest resistance to cotrimoxazole (38%) and the lowest resistance to amoxicillin/clavulanic acid (13%).

**Table 1:** Overall resistance rates and resistance rates of the most prevalent uropathogens

Uropathogen	Number positive cultures	Amoxicillin	Amoxicillin clavulanic acid	Ciprofloxacin	Trimethoprim	Cotrimoxazole	Nitrofurantoin	Fosfomycin
All uropathogens	105	64 (61%)	43 (41%)	36 (34%)	48 (46%)	(38%)	52 (50%)	19 (18%)
*E. coli*	40 (38%)	28 (70%)	20 (50%)	11 (28%)	10 (25%)	9 (23%)	2 (5%)	0 (0%)
Proteus	32 (30%)	11 (35%)	4 (13%)	10 (31%)	17 (53%)	12 (38%)	N/A^a^	3 (9%)

^a^Proteus mirabilis is intrinsically resistant to nitrofurantoin

### Reasons for obtaining urine cultures

**Fig. 3:**
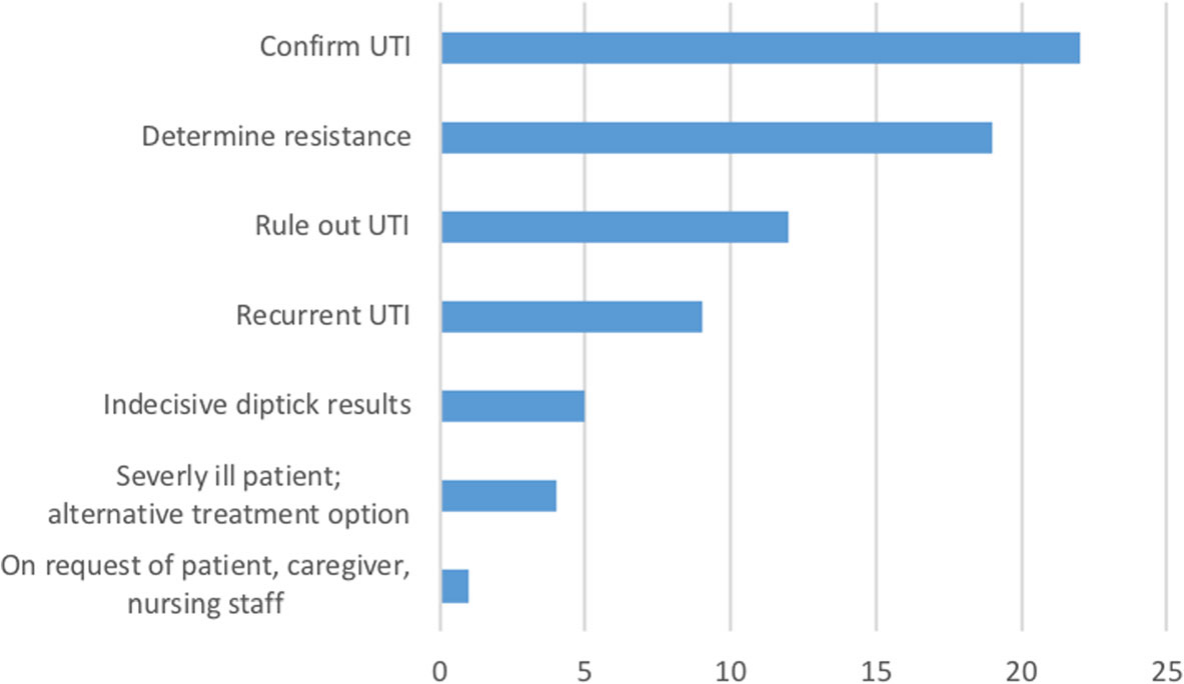
Reasons for obtaining urine culture

Seventy-two questionnaires were sent to the medical staff; of these, 59 were returned (82% response rate) and completed by all 13 physicians (three to seven per person). The most prevalent reason for all physicians to order a urine culture (*n* = 22) was to confirm the diagnosis of a UTI, to determine the AB resistance of the isolated microorganism (*n* = 19) and to rule out a UTI in 12 patients (Fig. 3).

The blank option was not used in any of the questionnaires.

Each physician mentioned at least once that a culture was requested to confirm the diagnosis of a UTI.

## Discussion

This study described the reasons for obtaining urine cultures and the actions taken based on the urine culture results in a LTCF.

In residents treated empirically with ABs for suspected UTIs, urine cultures were obtained in 8% of residents. In those cases in which the culture results suggested a reason for action, actions were taken in only 25%. The main reasons for ordering a urine culture were to confirm the diagnosis of a UTI, to determine the resistance of the isolated uropathogens or to rule out a UTI.

Several resident-related factors, diagnostic uncertainties and logistic problems might influence the diagnosis of a putative UTI and the decision regarding whether or not to take action according to culture results [20–23].

First, the infection might have been self-limiting, and complaints may have disappeared spontaneously or were no longer considered to be related to a UTI. In these situations, an initiation of or change in AB treatment are deemed unnecessary. We assume that if complaints remained at the time the culture results were reported, the nurses would have brought this issue to the attention of the responsible physician, and active follow-up would have been documented in the residents’ medical records.

Second, the high prevalence of ASB in LTCF residents [10, 12, 15, 21] and the non-specific signs and symptoms unrelated to UTIs, such as restlessness, aggression, ‘not being him or herself’ and drowsiness, are often considered to be falsely related to UTIs [10, 14–16, 24–26]. This finding is especially a problem in psychogeriatric care settings in which residents cannot always communicate their complaints properly [27]. In patients with dementia, behavioural problems have a fluctuating pattern during the course of the disease [28]. If physicians are convinced that behavioural problems in combination with ASB are caused by a UTI, AB treatment will be administered. Due to the fluctuating pattern of these behavioural problems, the symptoms often disappear spontaneously, suggesting that AB treatment was effective; this result might lead physicians to respond in the same manner in similar situations.

Third, AB treatment was continued in 93% of cases with negative culture results. Potentially, continuation of AB therapy was due to another reason, such as bacterial infections elsewhere in the body. However, indications for the continuation of AB treatment despite negative culture results were not found in the residents’ records.

In our study, treatment was not adapted to culture results in 75% of patients. Similarly, in an internal ward in a tertiary care hospital, when the culture results suggested a reason for action, treatment was not adjusted accordingly in 70% of cases [29]. Unfortunately, these authors did not explain the reasons for their findings.

In LTCFs, the prevalence of AB resistance is an increasing problem [30, 31]. In 2001, Loeb published the minimum criteria for initiation of ABs in nursing home residents to prevent inappropriate AB prescribing [13]. These criteria were based on expert opinion; since these criteria were published, no new useful studies have been performed to provide a higher standard of evidence. These criteria rely heavily on the presence of specific urinary tract symptoms (painful micturition, urgency, and frequency) and the presence of fever. Although these criteria are helpful in decreasing the misdiagnosis in UTIs in the general population, the presence of these specific complaints is often difficult to assess in psychogeriatric patients [27].

In the current study, the most frequently mentioned reason for ordering a urine culture was to confirm the diagnosis of a UTI. However, due to the high prevalence of ASB, a positive culture does not confirm the presence of a UTI [15, 20, 22].

Urine cultures are useful to rule out the diagnosis a UTI in cases of negative culture results and to obtain information about the AB susceptibility of the isolated microorganism to guide AB treatment [15].

Due to the logistical procedures in our LTCF and the microbiological laboratory, there was a long turnaround time for patients with positive culture results, ranging from three to twelve days. The duration from the time that the urine samples were obtained to the time they arrived at the laboratory, and the analysis was initiated ranged between one and three days.

The long turnaround time is due to logistical reasons. The LTCF sends the samples for microbiological analysis to the nearby hospital; then, the samples are sent to the regional microbiological laboratory located 35 km away. During weekdays/working hours, the samples are brought to the hospital daily by the faciliatory service of the LTCF and then to the laboratory. During the weekends, LTCF personnel must bring the samples to the hospital, usually at the end of their shifts. These samples are then sent to the laboratory for analysis the next working day. The results are sent to the hospital and then to the LTCF. Thus, a shorter turnaround time would positively influence the follow-up actions related to the culture results.

### Strength and weaknesses

To the best of our knowledge, this study is the first to evaluate urine culture results in relation to culture result-driven actions in a LTCF setting. A strength of this study was that the culture results were available in both patient records and as a printed version in both the physician’s mailbox and the ward’s mailbox. Thus, physicians did not miss the results.

A weakness of this study was its retrospective nature. We did not record the number of correct non-AB uses in patients with suspected ASB because our focus was on AB prescriptions for (putative) UTIs and urine cultures. Unfortunately, information regarding the motivations for and the reasons that physicians did not act according to the culture results were rarely recorded in patient charts. The lack of clinical information and information regarding the severity of the UTI episodes hindered a more accurate assessment of the reasons that physicians did not take action, i.e., to initiate, continue or change AB therapy.

The study was conducted in a single centre. Whether this medical team is representative of nursing home physicians in a non-academic setting in the Netherlands in terms of their diagnostic and treatment approach for putative UTIs is unknown, as is the applicability of the results of this study to other LTCFs. However, as the medical staff received their vocational training in different centres in the Netherlands, we expect that the results are also applicable to other LTCF settings. However, further studies in other LTCFs are warranted.

To improve the management of UTIs among residents of LTCFs, we suggest the following actions: i) reduce the turnaround time between when urine samples are obtained and culture results are reported; ii) increase the awareness of the medical staff to act according to culture results; and iii) increase the correct usage of urine cultures, specifically, for rejecting the diagnosis of a UTI when the culture results are negative and for obtaining information about the AB resistance of isolated microorganisms.

Optimisation of the diagnosis and treatment for putative UTIs in LTCF residents and adherence to guidelines are important and will contribute to the control of AB resistance problems in LTCFs. Recent guidelines recommend treating UTIs briefly (no longer than 7 days in women with febrile UTIs associated with pyelonephritis or 3–5 days in those with cystitis and only treating men with febrile UTIs lasting longer than 7 days); however, this treatment requires a turnaround time of no more than 5 to 7 days [32–34].

## Conclusions

In our LTCF, a urine culture was obtained in only 8% of the AB prescriptions for a putative UTI. In cases in which the urine culture results suggested a reason for action, action was taken in only 25%. The main reasons that doctors requested a urine culture was to confirm the diagnosis of a UTI and to obtain information about the AB resistance of the isolated uropathogens.

## Additional file

Additional file 1: Qstnr reasons for urine cultures. Questionaire about reasons for ordering urine cultures. (DOCX 15 kb)

## Abbreviations

AB: Antibiotic
AMR: Antimicrobial resistance
LTCF: Long-term care facility
UTI: Urinary tract infection
