# Supplementary material for: Urine cultures in a long-term care facility (LTCF): time for improvement
Source: BMC Geriatr. 2018 Sep 20;18:221. doi: 10.1186/s12877-018-0909-x (PMC6149184; doi:10.1186/s12877-018-0909-x)
Supplement: Supplementary file 1 — Qstnr reasons for urine cultures. Questionaire about reasons for ordering urine cultures. (DOCX 15 kb) [file 12877_2018_909_MOESM1_ESM.docx]

**Questionnaire: What are the reasons for ordering urine cultures in SZR?**

Date:

Patient number:

Name doctor:

**Questionnaire: What are the reasons for ordering urine cultures in SZR?**

Number questionnaire:

Dear Colleagues

As agreed upon in our medical team, we are performing a study on the reasons for ordering urine cultures. You receive this questionnaire because you have ordered a urine culture. The present date and patient number are mentioned on the first page. Please remove the first page before you hand in the completed questionnaire with the nurses of the medical secretariat. The information will be processed anonymously afterwards, although the number on this questionnaire can be used by the nurses to remind you in case you might forget to hand it in.

1. Please tick one or more reasons for ordering the urine culture below and if none of these apply, please fill in the blank space.

- Undecisive dipstick results
- To confirm the diagnosis UTI
- To rule out UTI
- To check whether previous antibiotic therapy has been effective
- Recurrent UTI
- To determine antibiotic resistance of uropathogens
- To provide alternative treatment options in case of a severely ill patient
- On request of patient or caregivers
- On request of nursing staff
- To buy some time so that I don’t have to start an antibiotic directly
- Other reason which is:

....................................................................................................................................

1. Have you empirically started an antibiotic (before the results will be available)?

- No
- Yes, I prescribed the following antibiotic: .......................................................

Thank you for your participation!

In case you have any questions, please contact Jobje Haaijman or the nurses at the medical secretariat.
